# Serial echocardiographic assessment of evolution of ductus arteriosus in preterm infants

**DOI:** 10.1590/S1679-45082014CE3052

**Published:** 2014

**Authors:** Bai-Horng Su

**Affiliations:** 1 Department of Neonatology, China Medical University Childrens Hospital, Taiwan, TW, China

Dear Sir,

I read with interest the recent article by Visconti et al.,^(1)^ describing the association of clinical and echocardiographic parameters and the evolution of the ductus arteriosus in preterm infants. The results of the patent ductus arteriosus (PDA) flow velocity, being higher in the cases in closing process and lower in the group with surgical closure, are similar to those described by us.^(2)^ However, unlike our study, no statistically significant difference in the PDA flow pattern was observed between the groups.

I agree with the statement by Visconti et al.^(1)^ that there is still no consensus on which echocardiographic parameters should be used to guide therapy. However, I would like to address the comment on this point. A recent study^(3)^ showed that ductal diameter and our PDA flow patterns^(2)^ are significantly associated, and PDA treatment decisions can be made with clinical efficacy and safety (fewer drug doses) when guided by assessment of ductal diameter or flow pattern. Furthermore, using both methods as a cross check against each other may enhance the clinical predictive capacity of echocardiography.

The serial echocardiographic assessment of PDA flow pattern can reflect the hemodynamic changes in PDA and predict the need for treatment with accuracy, as shown in our subsequent studies.^(4,5)^ Our PDA flow patterns are not only used to guide therapy, but also for withholding treatment. Although the pulmonary hypertension (PH) pattern has widest diameters,^(3)^ it is compatible with physiologically raised pulmonary pressures in the early life and also influenced by parenchymal diseases and their associated hypercapnia, alveolar hypoxia and acidosis. The foramen ovale and ductus arteriosus both serve to bypass the high resistance pulmonary vasculature during this PH status. Therefore surgeons should not try to close PDA of PH pattern for preventing further deterioration of elevated pulmonary hypertension. Effort should be focused on improving the lung condition to reduce the pulmonary artery pressures and hence decreasing PH, and keep watching for whether growing pattern or pulsatile pattern, the indicators for treatment of a significant PDA, will appear as the subsequent growing left-to-right shunting.

Finally, I would like to highlight the importance of the serial echocardiographic assessment of hemodynamic status rather than to depend only on a spot time measurement. What is most important is to know if the echocardiographic parameter can prospectively detect the development of a clinically significant PDA.
